# New Sex Chromosomes in Lake Victoria Cichlid Fishes (Cichlidae: Haplochromini)

**DOI:** 10.3390/genes13050804

**Published:** 2022-04-30

**Authors:** Thomas D. Kocher, Kristen A. Behrens, Matthew A. Conte, Mitsuto Aibara, Hillary D. J. Mrosso, Elizabeth C. J. Green, Michael R. Kidd, Masato Nikaido, Stephan Koblmüller

**Affiliations:** 1 Department of Biology, University of Maryland, College Park, MD 20742, USA; kbehren2@umd.edu (K.A.B.); conte1@gmail.com (M.A.C.); 2 Tokyo Institute of Technology, 2-12-1 Ookayama, Meguro-ku, Tokyo 152-8550, Japan; aibara.m.aa@m.titech.ac.jp (M.A.); mnikaido@bio.titech.ac.jp (M.N.); 3 Mwanza Fisheries Research Center, Tanzania Fisheries Research Institute (TAFIRI), Mwanza P.O. Box 475, Tanzania; hjmrosso@yahoo.com; 4 Department of Biology and Chemistry, Texas A&M International University, Laredo, TX 78041, USA; elizabethgreen@dusty.tamiu.edu (E.C.J.G.); michael.kidd@tamiu.edu (M.R.K.); 5 Institute of Biology, University of Graz, Universitätsplatz 2, 8010 Graz, Austria; stephan.koblmueller@uni-graz.at

**Keywords:** sex determination, sexually antagonistic selection

## Abstract

African cichlid fishes harbor an extraordinary diversity of sex-chromosome systems. Within just one lineage, the tribe Haplochromini, at least 6 unique sex-chromosome systems have been identified. Here we focus on characterizing sex chromosomes in cichlids from the Lake Victoria basin. In *Haplochromis chilotes,* we identified a new ZW system associated with the white blotch color pattern, which shows substantial sequence differentiation over most of LG16, and is likely to be present in related species. In *Haplochromis sauvagei,* we found a coding polymorphism in *amh* that may be responsible for an XY system on LG23. In *Pundamilia nyererei,* we identified a feminizing effect of B chromosomes together with XY- and ZW-patterned differentiation on LG23. In *Haplochromis latifasciatus,* we identified a duplication of *amh* that may be present in other species of the Lake Victoria superflock. We further characterized the LG5-14 XY system in *Astatotilapia burtoni* and identified the oldest stratum on LG14. This species also showed ZW differentiation on LG2. Finally, we characterized an XY system on LG7 in *Astatoreochromis alluaudi*. This report brings the number of distinct sex-chromosome systems in haplochromine cichlids to at least 13, and highlights the dynamic evolution of sex determination and sex chromosomes in this young lineage.

## 1. Introduction

Sex-chromosome turnover is rare in some lineages, such as mammals and birds, which have used the same chromosomes for sex determination for 180 and 100 MY, respectively [1,2]. However, in many other vertebrate lineages, different autosomes evolve into sex chromosomes frequently [3]. To understand the evolutionary mechanisms by which new sex chromosomes arise, we first need to characterize the rates and patterns of sex chromosome-turnover in these lineages.

Recent work has identified more than 22 unique sex chromosome systems on 18 of 23 chromosomes in East African cichlid fishes [4,5]. Within Haplochromini, the most species-rich cichlid tribe, which began to radiate within the last ~6 MY [6], sex determiners have been identified on at least 6 different chromosomes. XY systems have been identified on linkage groups (LGs) 7,13,18,19,23 and a fusion of LG5/14 [7,8,9,10,11]. ZW systems have been identified on LGs 5 and 13 [9,12]. B chromosomes have been associated with a quantitative effect on sex determination in Lake Victoria cichlids [13], and with a WO system in Lake Malawi cichlids [14]. Finally, a number of additional quantitative trait loci (QTL) for sex determination have been identified in cichlids from both Lake Victoria [15] and Lake Malawi [16].

A range of evolutionary forces might contribute to turnover of sex chromosomes, including genetic drift, natural selection on linked variation, and sex-ratio selection after environmental perturbation [17]. Evidence from Lake Malawi cichlids suggests that genetic conflicts are a frequent driver of sex-chromosome turnover. Sexually antagonistic selection on the cryptic orange-blotch color pattern recruited a dominant female sex determiner on LG5 [12]. Likewise, a B chromosome that exploits the asymmetric cell divisions of female meiosis recruited a dominant female sex determiner to promote its transmission across generations [14]. The evolution of female mouthbrooding in haplochromine cichlids may have increased the intensity of sexually antagonistic selection, and thereby accelerated the rates of sex-chromosome turnover in this lineage.

To further characterize the rates and patterns of sex-chromosome turnover in cichlids, and provide insight into the mechanisms driving sex-chromosome evolution, we performed a series of genome-wide association studies from the exceptionally young and species-rich Lake Victoria cichlid radiation (probably 15 ky, >500 endemic species) [18,19], plus species of *Astatotilapia* and *Astatoreochromis* from the Lake Tanganyika and Lake Victoria basins, respectively, that are closely related to the Lake Victoria superflock [20,21].

## 2. Materials and Methods

### 2.1. Fish and DNA Samples

Tissues were sampled from either unrelated individuals collected directly from the wild, or full-sib families reared in the lab. We followed the current taxonomy [22]. We sampled 28 normally colored males and 30 white-blotch females from a full-sib family of *Haplochromis (Paralabidochromis) chilotes*, a species found in rocky littoral habitats in Lake Victoria. We sampled 27 male and 18 female *Pundamilia nyererei*, and 32 male and 19 female *Haplochromis sauvagei,* directly from the wild at Kilimo Island (Mwanza Gulf, Tanzania). We analyzed 46 males and 25 females from a full-sib family of *Astatotilapia burtoni* derived from the Fernald–Hofmann laboratory strain originating from northern Lake Tanganyika. We also reanalyzed sequence data from previous publications on a lab strain of *Haplochromis (Astatotilapia) latifasciatus* [23,24]. Finally, we sampled 17 males and 26 females from a full-sib family of *Astatoreochromis alluaudi,* a molluscivore found throughout the Lake Victoria basin and the northern rift lakes.

### 2.2. Ethics Statement

Animal experiments were conducted in accordance with the Guide for Care and Use of Laboratory Animals. All animal use was approved under protocol numbers R-OCT-19-48 (U. Maryland), BMWFW-66.007/0004-WF/V/3b/2016 (U. Graz) and 2019-1 (TAMIU).

### 2.3. B Chromosome Screening and DNA Sequencing

DNA was purified from fin clips by phenol-chloroform extraction using phase-lock gel tubes (5Prime, Gaithersburg, MD, USA). DNA concentrations were quantified by fluorescence spectroscopy using a Quant-iT PicoGreen assay (ThermoFisher, Waltham MA, USA). Previous work indicated that *P. nyererei* shares B chromosome sequences with *H. latifasciatus* [23]. Therefore, we screened samples of *P. nyererei* and *H. sauvagei* for B chromosomes by qPCR of primer sets on scaffolds 13,19 and 26, using *HPRT* as a control (Table S1) [23]. Equimolar amounts of DNA from each individual were then pooled by sex for each species. Sequencing libraries were constructed, and 150 bp paired-end DNA sequencing was performed on a NovaSeq6000 S4 (Illumina, San Diego, CA, USA) by Novogene US (Davis, CA, USA) or the Maryland Genomics Center (University of Maryland, Baltimore, MD, USA).

### 2.4. Sequence Analysis

The sequence reads were aligned to the Nile tilapia (*Oreochromis niloticus*—UMD_NMBU, RefSeq GCF_001858055.2) and the Malawi zebra (*Maylandia zebra*—UMD2a, RefSeq GCF_000238955.4) assemblies [25,26] with BWA version 0.7.12 using the default parameters along with read group labels [27]. The alignments were sorted, marked for duplicates and indexed using Picard version 1.119 (http://picard.sourceforge.net accessed on 25 August 2014). Alignments were then converted into an mpileup file using Samtools version 0.1.18 and subsequently into a sync file using Popoolation2 [28,29]. Base calls with a PHRED score less than 20 were filtered out of the data set. We then used Sex_SNP_finder_GA.pl (https://github.com/Gammerdinger/sex-SNP-finder) to calculate *F*_ST_ between the male and female pools, and identify both XY- and ZW-patterned SNPs [30]. XY-patterned SNPs are defined as SNPs where one allele is fixed in the female pool, and the male pool is polymorphic for an alternate allele. ZW-patterned SNPs are defined as SNPs where one allele is fixed in the male pool, and the female pool is polymorphic for an alternate allele. The results were plotted in R using the plotrix package [31,32]. Read mappings in candidate regions were examined in IGV [33].

Although the *F*_ST_ and sexSNP plots are rich in detail, they are also subject to a variety of visual biases. To provide a stronger statistical foundation for our conclusions, we counted the number of sex-patterned SNPs in 100 kb windows across the genome using the bedtools2 window function [34]. We analyzed the top 1% of windows with the greatest number of XY- or ZW-patterned SNPs, and counted the number of windows, and the total number of sex-patterned SNPs in those windows, on each chromosome.

## 3. Results

### 3.1. Haplochromis chilotes—LG16 ZW

**Figure 1:**
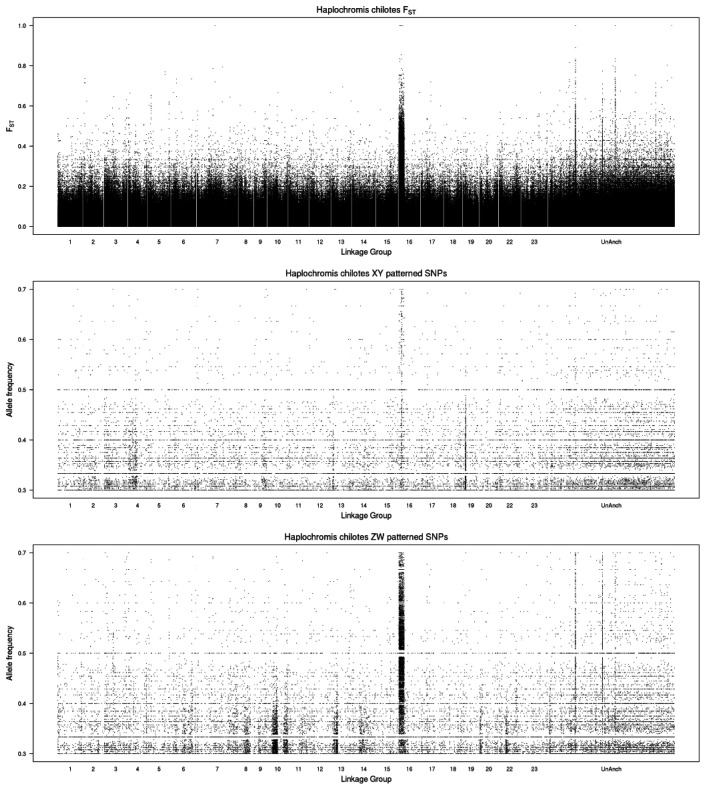
Whole genome plots comparing the male and female pools of *Haplochromis chilotes.* The **top** panel shows values for *F*_ST_. The **middle** panel shows the frequency of Y-patterned SNPs in the male pool. The **bottom** panel shows the frequency of W-patterned SNPs in the female pool. The reference genome is the *Maylandia zebra* UMD2a assembly.

The whole genome plot of differentiation mapped on the *M. zebra* assembly identifies a ZW system on LG16 (Figure 1). Three unanchored scaffolds (000524F, 000797F, 000914F) also show ZW signals, and likely belong to LG16. When mapped on the more contiguous *O. niloticus* assembly, all of the differentiation falls on LG16 (data not shown).

**Figure 2:**
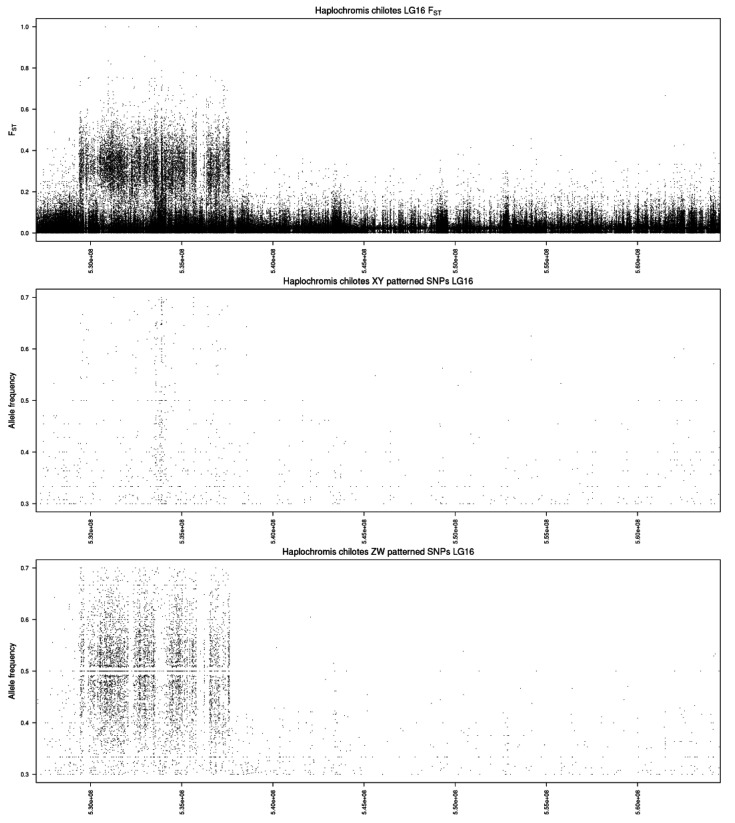
Single chromosome plot for *Haplochromis chilotes.* The **top** panel shows values for *F*_ST_. The **middle** panel shows the frequency of Y-patterned SNPs in the male pool. The **bottom** panel shows the frequency of W-patterned SNPs in the female pool. The x-axis is labeled with the coordinates of the *Maylandia zebra* UMD2a reference assembly.

The single chromosome plot for LG16 (Figure 2) shows that the differentiation spans roughly 8.4 Mb near the beginning of LG16 (0.9–9.3 Mb). We hesitate to suggest candidate genes within this large region. The number of sex-patterned SNPs is uniform, except for the region from 5.1 to 5.8 Mb, which shows an excess of XY-patterned SNPs. This may represent an evolutionary stratum that has lost many sequences from the W, causing polymorphisms on the Z chromosome to be detected as XY-patterned SNPs. This region is annotated with several olfactory receptors and fragments of uncharacterized genes. Annotations for the three unanchored scaffolds do not contain any obvious candidate genes. Despite the young age of the LG16 system in *P. chilotes*, the differentiation between the sex chromosomes extends over many megabases. Aside from the possible evolutionary stratum from 5.1–5.8 Mb, the differentiation is uniform and thus may have originated in a single event of structural mutation (e.g., chromosomal inversion).

There is also an intriguing XY signal on LG19, which might represent a second sex determiner segregating in this family. The single chromosome plot for LG19 (Figure S1) shows XY-patterned SNPs in the region from 4.1 to 5.2 Mb. Genes in this region include the estrogen receptor *esr2* (4.19 Mb) [35], and *jag2* (4.43 Mb) which is involved in notch signaling in the testis [36].

**Figure 3:**
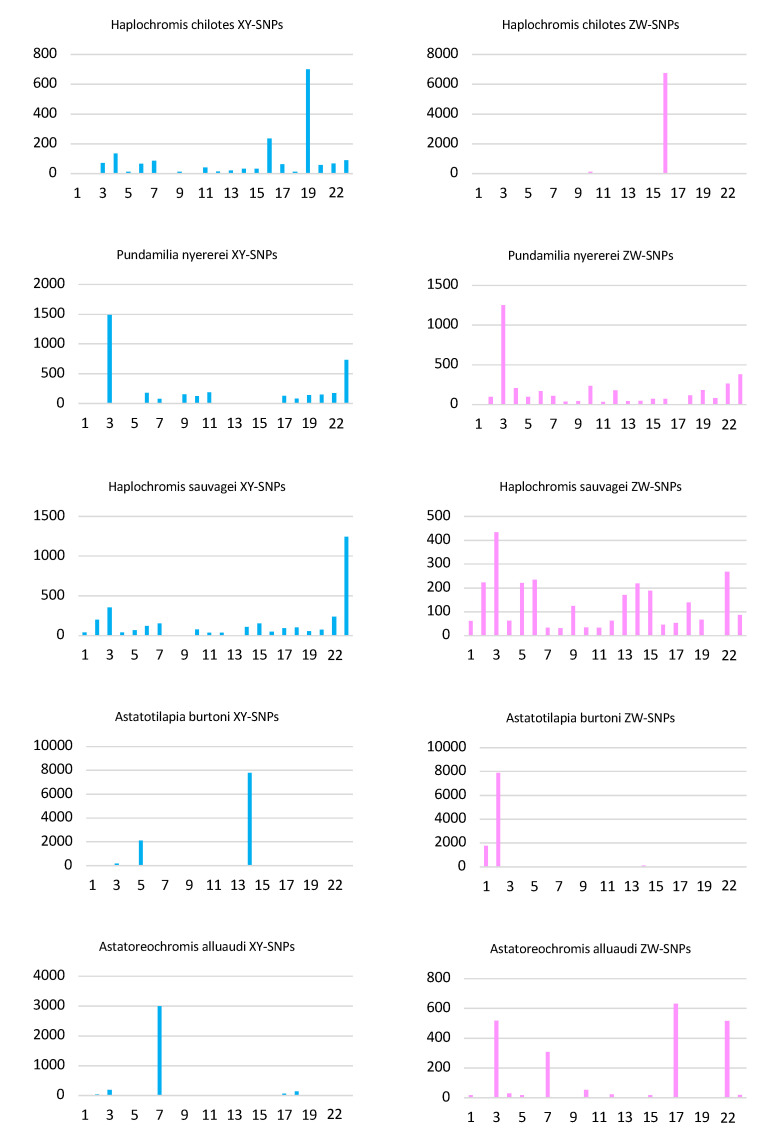
Analysis of sex-patterned SNPs in 100 kb windows. The number of SNPs in the top 1% of windows are plotted for both XY- and ZW-patterned SNPs in each of the five species. The *x*-axis represents linkage groups in the *Maylandia zebra* UMD2a assembly.

The analysis of the top 1% of 100 kb windows identified 65 windows with a total of 6733 ZW-patterned SNPs on LG16 (Figure 3 and Table S2). It also identified 13 windows with a total of 700 XY-patterned SNPs on LG19. An additional 8 windows with 236 XY-patterned SNPs were found on LG16, and probably represent regions of ZZ heterozygosity in regions missing from the W.

### 3.2. Pundamilia nyererei—Feminizing B and LG23 XY

Each DNA sample was screened for B chromosomes with the qPCR assays for three B-specific fragments. We made separate DNA pools for the 18 females with B chromosomes, 14 males with B chromosomes and 13 males without B chromosomes. Since all of the females but only half of the males had B chromosomes, these results suggest a feminizing effect of B chromosomes in this population.

Whole genome sequencing was performed separately on each of these groups, and comparisons were made between the males with B chromosomes and the females with B chromosomes (Figure S2). On the UMD2a assembly, there is weak differentiation across the first half of LG23 (Figure S3). There are more XY-patterned than ZW-patterned SNPs (Figure 3). Within this region, we identified four peaks of differentiation. The first peak (4.70–4.76 Mb) includes *gzmb*, a granzyme B/G-like protease that is expressed in mammalian granulosa cells and affects apoptosis, and *yjefn3,* which is involved in steroid metabolism [37,38]. The second peak (16.7–17.1 Mb) contains *gli2*, a gene involved in hedgehog signaling and the development of Leydig cells in mice [39]. The third peak (17.5–18.0 Mb) contains *sh3yl1*, whose product binds to the proline-rich N-terminus of the androgen receptor to regulate androgen-mediated cell growth and migration [40]. A fourth peak (20.5 Mb) is centered on *nlrc5*, a member of the NACHT nucleoside triphosphatase (NALP/NRLP) family involved in a variety of gonadal phenotypes [41,42]. Variation in the gene for anti-Mullerian hormone (Amh), a ligand activating the TGF-ß pathway, has been implicated in sex determination in many fish species [43]. We found relatively little SNP differentiation around the *amh* gene at 11.1 Mb, and no amino acid sequence variation. However, there were at least two male-associated structural variants near *amh* identified by examining the mappings of paired reads in IGV (Figure S5). First, there is a 112 kb inversion (11.018–11.130 Mb on *M. zebra* UMD2a) centered on *amh* which is found almost exclusively in males with or without B chromosomes. Second, there is a 6 kb duplication encompassing most of *fkbp8* (11.020 Mb), which is found exclusively in males. Both males and females have a 6 kb duplication in the first intron of *ell* (11.040 Mb).

The analysis of 100 kb windows also identified a large number of XY-patterned SNPs widely distributed across LG3 (Table S2). We were not able to identify any discrete peaks of differentiation on this chromosome (Figure S3). We did not see any evidence of the LG9 ZW locus reported in related species of *Pundamilia* [44], which perhaps indicates the diversity of sex-determining mechanisms in this genus.

### 3.3. Haplochromis sauvagei—LG23 XY

qPCR of the three B-specific fragments and an analysis of sequence coverage at *ihhb* [13] showed no evidence of a B chromosome in *H. sauvagei*. The whole genome plot suggests an XY signal on LG23 (Figure S6), and this is supported by the analysis of 100 kb blocks (Figure 3, Table S2). The peak of differentiation (7.5–15.5 Mb) is centered on *amh* at 11.1 Mb (Figure S7). The read coverage in this region provides no indication of sex-specific structural variation at *amh*. However, there is a V489L substitution near the end of the coding sequence at 11,102,421 bp in males. This site is homologous to human Amh L536, which is in the ß6 domain that makes up part of the interface between Amh and its receptor [45].

The analysis of 100 kb windows also suggests weak ZW-patterned differentiation on LG3 (Figure 3). Thirteen of the 74 top 1% bins are from LG3 (a 3.6-fold enrichment) and they contain 433 ZW-patterned SNPs (Table S2). However, these ZW-patterned SNPs are distributed along most of LG3, and do not suggest any candidate genes.

### 3.4. Haplochromis latifasciatus—Duplication of AMH

The frequent involvement of *amh* in teleost sex determination caused us to reanalyze published whole genome sequence data for *H. latifasciatus* [23,24]. B chromosomes have been identified in this species, but are found in similar frequencies in both males and females [46]. We mapped the reads to *M. zebra* UMD2a and identified a 9205 bp tandem duplication of *amh*, with breakpoints at 11,096,836 and 11,106,041 bp on LG23 (Figure S8). The duplication is present in 3 of 4 males, and is not present in the single female examined (Table 1). Our limited sample did not allow us to determine whether this duplication is associated with sex. However, given the many reports of *amh* variation linked to sex determination in fishes, this polymorphism should be examined further in *H. latifasciatus* and in related species in Lake Victoria.

### 3.5. Astatotilapia burtoni—LG5/14 XY

The whole genome plot on the *M. zebra* UMD2a reference shows strong differentiation consistent with an XY system across most of LG5 and LG14 (Figure S9). The overall pattern of differentiation is similar using either the *M. zebra* or *O. niloticus* reference assemblies, and suggests a series of evolutionary strata associated with structural rearrangements, including a fusion of LG5 and LG14.

The sharp *F*_ST_ peak at 17.5–17.7 Mb on LG5 is reflected in the XY SNP plot, and contains one uncharacterized protein (LOC111500363; Figure S10). The homologous region of *O. niloticus* (20.93–20.98 Mb) is annotated with two uncharacterized proteins (LOC102080880, LOC112846922) that are highly expressed in testis [47]. There is evidence for structural polymorphisms throughout this region, but none seem to be sex-specific. The segment with the largest number of differentiated SNPs is on LG14 (29–32 Mb on *M. zebra*) (Figure S11), but this region contains no obvious candidate genes for sex determination.

Surprisingly, there is equally strong ZW-patterned differentiation on LGs 1 and 2 in this family (Figure S12 and Figure S13). There are 1759 ZW-patterned SNPs on LG1, and 7868 on LG2, among the top 1% windows (Figure 3, Table S2). There are no obvious peaks within these relatively large blocks of differentiation.

### 3.6. Astatoreochromis alluaudi—LG7 XY

The whole genome plot identifies two blocks of XY-patterned differentiation on LG7 (Figure S10). The first block extends from 47.5–53.0 Mb on the *M. zebra* UMD2a assembly (Figure S15). The lower boundary is close to the candidate gene *gsdf* (47.495 Mb), but there is no indication of coverage or sequence differentiation in this gene. *lam3c* (49.65 Mb), does show SNP differentiation and encodes laminin gamma 3 expressed in Leydig cells [48]. *btf3* (51.170 Mb) shows numerous differentiated SNPs and is associated with gonadal phenotype in chicken [49]. *foxd1* (51.179 Mb) is also differentiated and has a possible role in testis development [50]. The second block of differentiation on LG7 extends from 58.25–63.5 Mb. Differentiation is strongest at 58.5 Mb, but none of the genes in the vicinity have an obvious link to gonad development.

The analysis of 100 kb blocks identifies ZW-patterned differentiation on LGs 3, 7, 17 and 22 (Figure 3; Table S2). A sharp peak of ZW signal on LG7 at 20 Mb is centered on *cspg4*, a pericyte marker expressed in the ovarian thecal layer [51]. None of the other high-scoring blocks contain obvious candidate genes for sex determination.

## 4. Discussion

**Figure 4:**
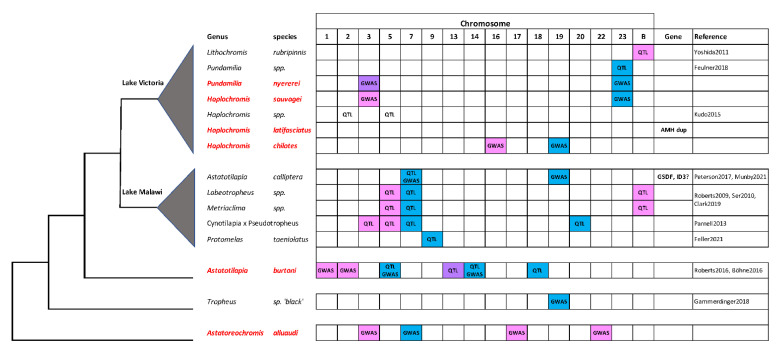
Sex chromosomes in the Haplochromini. The phylogenetic relationships of some haplochromine species are shown on the left. The species studied in this paper are listed in red. Blue boxes indicate XY systems, pink boxes indicate ZW systems, and purple boxes indicate instances of XY and ZW variation on the same chromosome in *P. nyererei* and *A. burtoni*. Support from QTL or genome wide association studies (GWAS) is indicated, and candidate genes are listed to the right. Yoshida et al., 2011 [13], Feulner et al., 2018 [10], Kudo et al., 2015 [15] Peterson et al., 2017 [53], Munby et al., 2021 [54]; Roberts et al., 2009 [7], Ser et al., 2010 [12], Clark et al., 2019 [14]; Parnell et al., 2013 [16]; Feller et al., 2021 [44]; Roberts et al., 2016 [8], Böhne et al., 2016 [9]; Gammerdinger et al., 2018 [11].

Cichlid fishes in the subfamily Pseudocrenilabrinae have radiated from a common ancestor ~10 MYA to produce the more than 1000 species of the East African radiation (EAR) [6,19]. Previous work has implicated at least 12 of 23 linkage groups as sex chromosomes in at least one species of the EAR [5]. The present work contributes to the identification of sex loci on a total of 13 chromosomes in the haplochromine sub-lineage alone (Figure 4). These results clearly demonstrate that a large number of genes have the potential to become sex determiners in cichlid fishes.

### 4.1. A New Sex Chromosome on LG16

We identified a new locus on LG16 as the sex determiner in *H. chilotes*. LG16 has not been identified previously as a sex chromosome in any East African cichlid. The association of the white-blotch polymorphism with sex is reminiscent of the association of the orange-blotch polymorphism with a ZW sex determiner on LG5 in Lake Malawi cichlids [12]. It will be interesting to learn if sexual antagonism over color pattern played a role in the evolution of the LG16 ZW system in Lake Victoria.

### 4.2. B Chromosomes

Our data suggest a feminizing effect of B chromosomes in *P. nyererei*. This is consistent with the reported feminizing effect of B chromosomes in *Lithochromis rubripinnis* from Lake Victoria [13] and in several species of Lake Malawi cichlids [14]. The now frequent association of B chromosomes with sex suggests an important role for genetic conflicts in the evolution of sex determination. There is a fascinating intersection of evolutionary arms races involving selfish genetic elements, sex determination and sexually antagonistic selection, which deserves further investigation.

### 4.3. Amh

Our data suggest that polymorphisms in *amh* may play an important role in XY systems in Lake Victoria cichlids. We identified two possible Y alleles near *amh* in *Pundamilia*, and a potentially functional amino acid polymorphism of Amh in *H. sauvagei*. We also identified a duplication of *amh* on LG23 in *H. latifasciatus*. At this point it is not clear whether this duplication impacts sex determination, but since this gene plays an important role in sex determination in other fishes, it deserves further investigation across the broader Lake Victoria flock.

### 4.4. Astatotilapia burtoni

An XY sex-determination system in *A. burtoni* was first inferred through hormonal reversal and production of monosex offspring [52]. Subsequent efforts to localize the sex determiner have produced varying results, depending on the particular strain of *A. burtoni* examined. RAD sequencing identified an XY system involving a fusion of LG5 and LG14 in strains from northern Lake Tanganyika [8,9]. An additional LG13 WXY system was identified segregating in one family of the northern strain [9], while an additional LG18 XY system was found in fish from southern Lake Tanganyika [8]. The WGS data in the current paper confirm the LG5-14 XY system in a northern strain, identify new sex loci on LG2 and suggest a relatively long and complex history for the sex chromosomes in this species. Long-read sequencing and haplotype-resolved assemblies will be needed to make further progress on this system.

## 5. Conclusions

For a long time, sex chromosomes have been considered typologically—we assumed that each species segregated a single sex-chromosome system and that sex-chromosome turnovers were rare. Recent work, however, has revealed the fractal nature of the genetic variation for sex determination. The closer we look, the more polymorphisms affecting sex determination we find. In haplochromine cichlids, it is now usual to find multiple sex determiners within species, and even within a locus we may find multiple alleles [54].

The radiation of the Lake Victoria species flock ‘sensu stricto’ is probably only 15 ky old, while the entire ‘superflock’ inhabiting the Lake Victoria basin is only 10 times older [18]. It is thus astounding to find so many sex determiners segregating in the limited sample of species that have been studied so far. There appear to be several XY systems based on different variants of *amh*. There are female-biasing (W) factors located on B chromosomes. There is a ZW system linked to white-blotch on LG16, and an additional ZW system linked to orange-blotch [12,55]. Finally, there is evidence for sex-determining loci on LG2 and LG5 from a QTL cross between an *H. chilotes* female (presumably ZW on LG16) and an *H. sauvagei* male (presumably XY on LG23) [15]. There is little doubt that future research will uncover additional sex-determining loci among Lake Victoria cichlids.

This diversity begs an explanation. The large number of sex determiners might have arisen in separate lineages prior to the hybridization events that are thought to have been foundational for the Lake Victoria radiation [18]. High levels of intraspecific polymorphism might reflect continuing introgressive hybridization among species with different sex-determining systems. However, these explanations depend on a generally high rate of sex-chromosome turnover in the haplochromine lineage to create the diversity that is mixed via hybridization.

Alternatively, the diversity of sex determiners may arise from ongoing genetic conflicts among different genomic compartments. These compartments include B chromosomes undergoing meiotic drive and the non-recombining regions around sex determiners experiencing sexually antagonistic selection. The highly differentiated sex roles of these maternal mouthbrooding cichlids may create sexually antagonistic selection on hundreds of loci across the genome, creating opportunities for the invasion of new sex determiners. The complexity of these polygenic systems may allow the persistence of these polymorphisms for considerable periods of time.

In any case, these high levels of polymorphism make cichlids an ideal system for studying the population genetics of sex-chromosome turnover. Future population genomic studies may reveal the detailed evolutionary dynamics of sex determination and its interaction with the genetics of speciation in this extraordinarily biodiverse lineage [56,57].

## Figures and Tables

**Table 1:** Occurrence of an *amh* gene duplication in *H. latifasciatus* from whole genome sequences.

GenBank Accession	B Chromosome Content	Amh Duplication
Female—SRX10474226	1 B	No
Male—SRX2530877	2 B	No
Male—SRX2530878	0 B	Yes
Male—SRX10474227	1 B	Yes
Male—SRX10474228	1 B	Yes

## Data Availability

All sequence reads have been deposited in the NCBI SRA under Bioproject PRJNA802233 (https://www.ncbi.nlm.nih.gov/bioproject/PRJNA802233).

## Supplementary Materials

The following supporting information can be downloaded at https://www.mdpi.com/article/10.3390/genes13050804/s1. Figure S1: *Haplochromis chilotes* Chr19 on *M. zebra* UMD2a reference assembly; Figure S2: *Pundamilia nyererei* whole genome on *M. zebra* UMD2a reference assembly; Figure S3: *Pundamilia nyererei* Chr23 on *M. zebra* UMD2a reference assembly; Figure S4: *Pundamilia nyererei* Chr3 on *M. zebra* UMD2a reference assembly; Figure S5: Structural variants near AMH in *Pundamilia nyererei*; Figure S6: *Haplochromis sauvagei* whole genome on *M. zebra* UMD2a reference assembly; Figure S7: *Haplochromis sauvagei* Chr23 on *M. zebra* UMD2a reference assembly; Figure S8: Duplication of AMH on *Haplochromis latifasciatus* LG23; Figure S9: *Astatotilapia burtoni* whole genome on *M. zebra* UMD2a reference assembly; Figure S10: *Astatotilapia burtoni* Chr5 on *M. zebra* UMD2a reference assembly; Figure S11: *Astatotilapia burtoni* Chr14 on *M. zebra* UMD2a reference assembly; Figure S12: *Astatotilapia burtoni* Chr1 on *M. zebra* UMD2a reference assembly; Figure S13: *Astatotilapia burtoni* Chr2 on *M. zebra* UMD2a reference assembly; Figure S14: *Astatoreochromis alluaudi* whole genome on *M. zebra* UMD2a reference assembly; Figure S15: *Astatoreochromis alluaudi* Chr7 on *M. zebra* UMD2a reference assembly. Table S1: qPCR primers for B chromosomes; Table S2: Analysis of 100 kb windows in five species of haplochromine cichlids.
