# Supplementary figures and images for: New Sex Chromosomes in Lake Victoria Cichlid Fishes (Cichlidae: Haplochromini)
Source: Genes (Basel). 2022 Apr 30;13(5):804. doi: 10.3390/genes13050804 (PMC9141883; doi:10.3390/genes13050804)

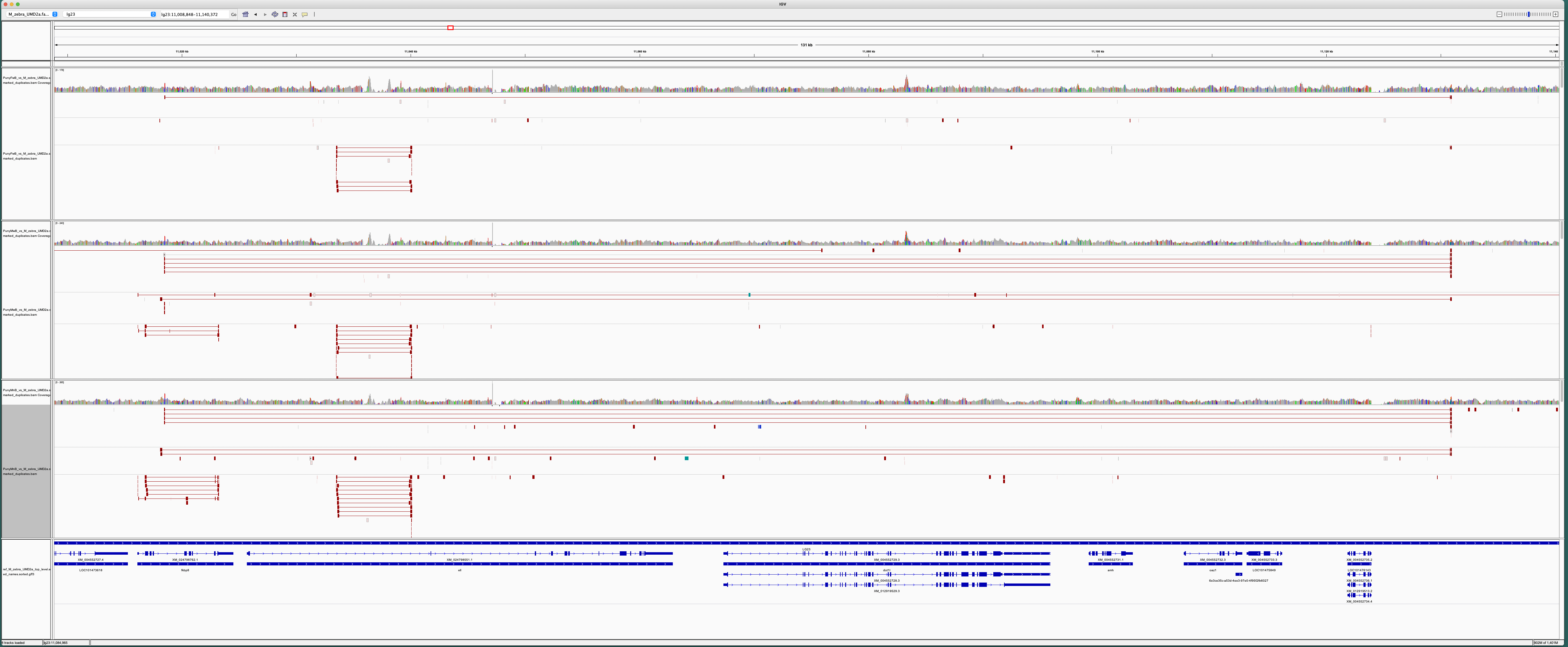

Supplement: Supplementary file 1 [file genes-13-00804-s001.zip › genes-1674854-suppl/Figure S5.png]

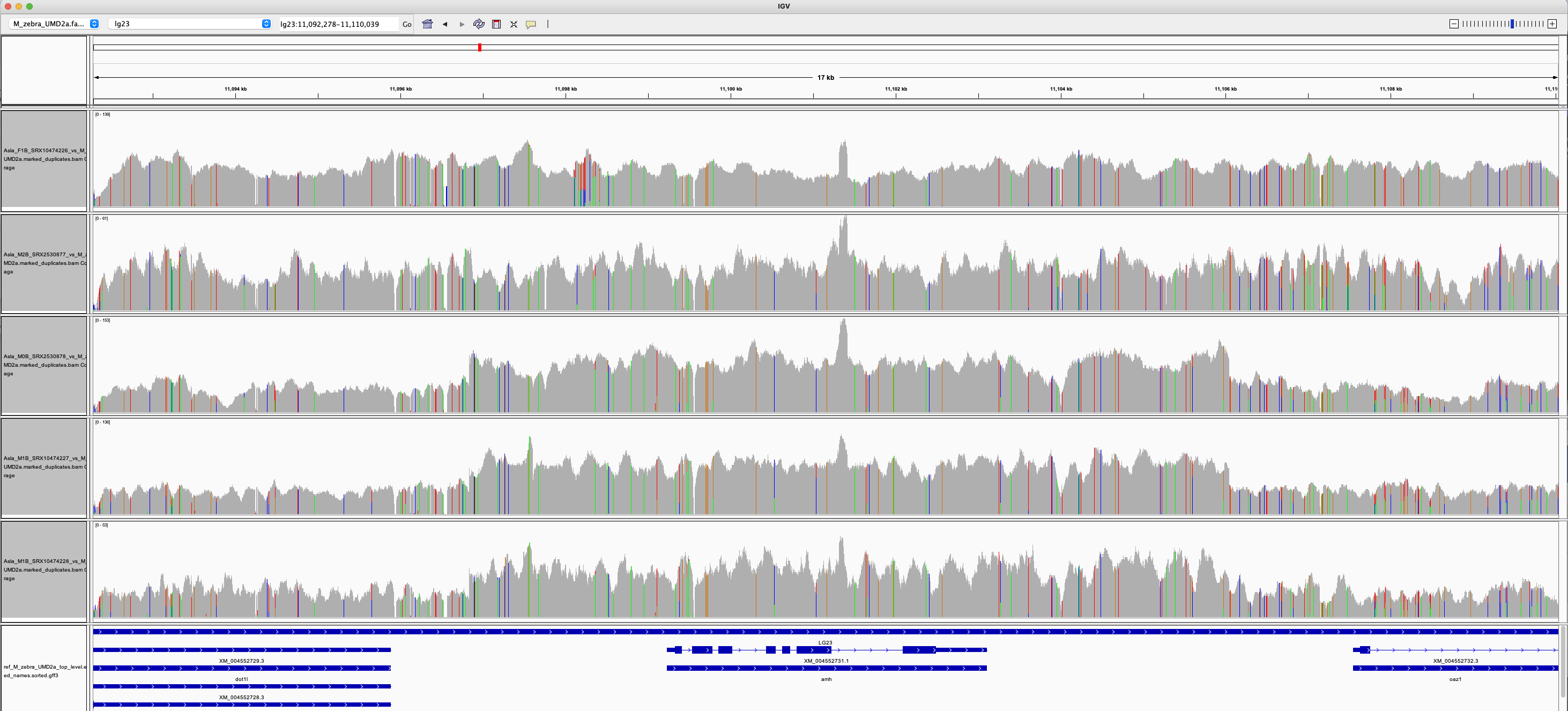

Supplement: Supplementary file 1 [file genes-13-00804-s001.zip › genes-1674854-suppl/Figure S8.png]
